# Approaching the Functional Annotation of Fungal Virulence Factors Using Cross-Species Genetic Interaction Profiling

**DOI:** 10.1371/journal.pgen.1003168

**Published:** 2012-12-27

**Authors:** Jessica C. S. Brown, Hiten D. Madhani

**Affiliations:** Department of Biochemistry and Biophysics, University of California San Francisco, San Francisco, California, United States of America; The University of North Carolina at Chapel Hill, United States of America

## Abstract

In many human fungal pathogens, genes required for disease remain largely unannotated, limiting the impact of virulence gene discovery efforts. We tested the utility of a cross-species genetic interaction profiling approach to obtain clues to the molecular function of unannotated pathogenicity factors in the human pathogen *Cryptococcus neoformans*. This approach involves expression of *C. neoformans* genes of interest in each member of the *Saccharomyces cerevisiae* gene deletion library, quantification of their impact on growth, and calculation of the cross-species genetic interaction profiles. To develop functional predictions, we computed and analyzed the correlations of these profiles with existing genetic interaction profiles of *S. cerevisiae* deletion mutants. For *C. neoformans LIV7*, which has no *S. cerevisiae* ortholog, this profiling approach predicted an unanticipated role in the Golgi apparatus. Validation studies in *C. neoformans* demonstrated that Liv7 is a functional Golgi factor where it promotes the suppression of the exposure of a specific immunostimulatory molecule, mannose, on the cell surface, thereby inhibiting phagocytosis. The genetic interaction profile of another pathogenicity gene that lacks an *S. cerevisiae* ortholog, *LIV6*, strongly predicted a role in endosome function. This prediction was also supported by studies of the corresponding *C. neoformans* null mutant. Our results demonstrate the utility of quantitative cross-species genetic interaction profiling for the functional annotation of fungal pathogenicity proteins of unknown function including, surprisingly, those that are not conserved in sequence across fungi.

## Author Summary

HIV/AIDS patients, cancer chemotherapy patients, and organ transplant recipients are highly susceptible to infection by opportunistic fungal pathogens, organisms common in the environment that are harmless to normal individuals. Understanding how these pathogens cause disease requires the identification of genes required for virulence and the determination of their molecular function. Our work addresses the latter problem using the yeast *Cryptococcus neoformans*, which is estimated to cause 600,000 deaths annually worldwide in the HIV/AIDS population. We describe a method for determining gene function in which *C. neoformans* genes are expressed in deletion mutants of all nonessential genes of the well-studied model yeast *S. cerevisiae*. By examining the impact on growth (enhancement or suppression) we generated “cross-species” genetic interaction profiles. We compared these profiles to the published genetic interaction profiles of *S. cerevisiae* deletion mutants to identify those with correlated patterns of genetic interactions. We hypothesized that the known functions of *S. cerevisiae* genes with correlated profiles could predict the function of the pathogen gene. Indeed, experimental tests in *C. neoformans* for two pathogenicity genes of previously unknown function found the functional predictions obtained from genetic interaction profiles to be accurate, demonstrating the utility of the cross-species approach.

## Introduction

Diseases produced by fungal infections are challenging to diagnose and treat, making these infections a major cause of morbidity and mortality worldwide [1], [2]. Genetics and genomics have led to the identification of numerous pathogen genes required for replication in the mammalian host [3]–[7]. Unfortunately, many, if not most, identified virulence genes lack *in vitro* phenotypes that could explain their effects in the host [3]–[8], and the predicted protein sequences often offer few clues to function. Thus, our power to identify pathogen genes required for disease far outstrips our ability to understand their molecular function in the host.

Historically, the expression of human genes in the model yeasts *Saccharomyces cerevisiae* and *Schizosaccharomyces pombe* has been used as a tool to identify specific genes and to determine their cellular function [9]–[14]. In a classic example, complementation of a fission yeast *cdc2* mutant was used to identify human Cdk1 [11]. More recently, a number of groups have combined the expression of foreign genes with high-throughout tools available in *S. cerevisiae* to identify suppressor genes to obtain insights into the function of human proteins, ranging from those involved in neurodegeneration to cancer [9], [11], [14].

Likewise, expression of viral and bacterial proteins in yeast, coupled with subsequent genetic analysis, has proven to be informative. For example, the genes responsible for biosynthesis of the eEF2 modification diphthamide were identified in selections for resistance to the F2 fragment of diphtheria toxin [15]. Identification of *S. cerevisiae* gene deletion mutants hypersensitive to the expression of the *Shigella* virulence factor OspF, a type III secretion substrate, coupled with transcriptional profiling experiments, led to the identification of the cell wall integrity MAP kinase pathway as a likely target of OspF in yeast [10], [13]. Importantly, the latter study took advantage of phenotypic information for yeast deletion mutants available at that time to obtain clues to gene function [10].

The construction of a library of all nonessential gene deletions for *S. cerevisiae* [8] together with the development of genetic selections led to the development of the synthetic genetic array (SGA) method for quantitatively measuring genetic interactions on a genome scale [16], [17]. This approach has facilitated the systematic annotation of gene function in *S. cerevisiae* [18], [19]. Genetic interaction, or epistasis, measures the degree to which two genes affect each other [16], and is measured by comparing the phenotype of a double mutant to that of the two corresponding single mutants. Genes that act in the same pathway display similar patterns of genetic interactions with other genes [16]–[19]. Recently, the large-scale application of these methods led to production of a remarkable genome-scale genetic interaction map based on the analysis of ∼5.4 million gene pairs. Such a comprehensive genetic interaction dataset has only been described to date for the model yeast *S. cerevisiae* [19].

Below we test the utility a cross-species genetic interaction approach for fungal pathogen gene annotation that combines expression of pathogen genes in *S. cerevisiae* with genetic interaction profiling. We used genes from the human pathogen *Cryptococcus neoformans*, an opportunistic basidiomycete fungal pathogen that is very distantly related to the model yeasts *S. cerevisiae* and *S. pombe. C. neoformans* is the most common cause of fungal meningitis in humans, and among the most important causes of morbidity and mortality in AIDS patients, leading to ∼1 million infections and ∼600,000 deaths annually in sub-Saharan African alone [1]. Our laboratory previously generated a library of 1201 gene deletion strains and used a signature-tagged mutagenesis approach to identify genes required for pathogen fitness during experimental infection of mice [5]. In addition to identifying new genes required for the synthesis of known virulence factors, these studies identified several dozen genes required for virulence whose mutation failed to yield *in vitro* phenotypes that could explain its role in the host.

As a proof-of-principle, we expressed six *C. neoformans* genes of interest in each member of the *S. cerevisiae* deletion library and quantified their impact on fitness, thereby producing cross-species genetic interaction profiles. We exploited their similarities to existing *S. cerevisiae* knockout genetic profiles to predict possible functions for each *C. neoformans* protein. For two of these *C. neoformans* proteins, Liv6 and Liv7, we describe validation experiments that support the functional assignment. For Liv7, additional experiments connect its newly identified function to the evasion of phagocytosis, an important virulence trait. The cross-species genetic interaction profiling approach described here offers a generalizable avenue toward the functional annotation of pathogenicity factors of fungal agents of infectious disease.

## Results

### Cross-species genetic interaction approach

**Figure 1:**
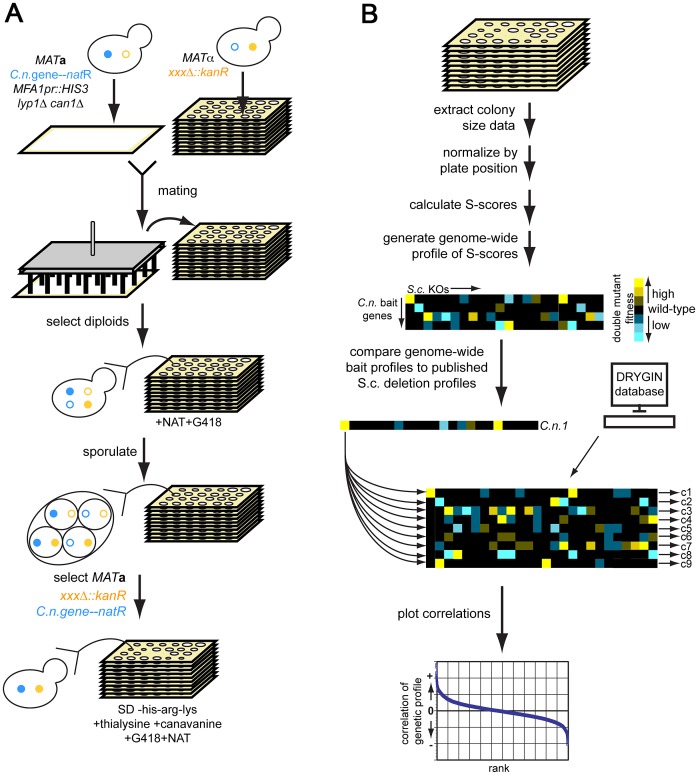
Cross-species genetic interaction mapping to predict the function of *C. neoformans* genes necessary for murine infection. A) Cross-species genetic interaction method. We created *S. cerevisiae* strains expressing each *C. neoformans* gene of unknown function at the *URA3* locus under the control of the *S. cerevisiae GPD1* promoter [77] and linked to nourseothricin (NAT) resistance. We used the synthetic genetic analysis (SGA) strain background, which allows for selection of the *MAT*a mating type and, ultimately, the haploid cellular state [16], [17]. We crossed this strain to the *S. cerevisiae* deletion library of targeted gene deletions marked by kanamycin (G418) resistance [8]. We selected for diploids on YPAD+NAT+G418, then sporulated diploids on sporulation medium, selected for *MAT* **a** haploids (+thialysine+canavanine), and then selected for the *C. neoformans* gene expression construct and the knockout mutations (+NAT+G418+thialysine+canavanine). See Methods and references 10–11 for description of the SGA method. B) Analysis of cross-species genetic interaction data. We scanned plates with colonies containing both the *C. neoformans* expression construct and the *S. cerevisiae* knockout mutations with a flatbed scanner. We extracted colony size information using ScreenMill [76], then normalized colony size data using the S-score method [20]. We generated an S-score for each double mutant strain (*C.n.* expression construct combined with knockout mutant), then computed the Pearson correlation between each such profile and genome-wide profiles available for *S. cerevisiae* gene deletions [19]. We converted the correlations to Z-scores and filtered out hits for which the Z-score of either the vector or the GFP control was ≥1.96 (p = 0.05). We also filtered out hits whose ratios of *C. neoformans* gene correlation score/control correlation score (either vector or GFP) was between 0.95 and 1.05 (∼60 profiles).

We sought to develop a generic approach for developing testable hypotheses for the function of novel *C. neoformans* virulence genes by taking advantage of the methods and datasets that exist in *S. cerevisiae*. We created *S. cerevisiae* strains that each expressed a *C. neoformans* gene of interest (described further below). We crossed these to the *S. cerevisiae* gene deletion library using automated SGA methods and measured fitness of the progeny strains using high-throughput colony imaging methods [16], [17] (Figure 1). Measurements (n = 8 per double mutant) were converted into significance scores (S-scores) [20] (See Methods). We refer to these data as a “cross-species genetic interaction profile” which is the set of quantitative genetic interactions between strains expressing a particular *C. neoformans* gene and each *S. cerevisiae* deletion mutant. We calculated correlations between these cross-species profiles and the available genetic interaction profiles of deletion mutants in *S. cerevisiae* [21]. We reasoned that the expression of a *C. neoformans* gene could, in some cases, produce dominant-negative effect and produce genetic interaction profiles that correlate positively with those of *S. cerevisiae* gene deletions that function in the homologous pathway. Alternatively, the expression *C. neoformans* gene might have a dominant-positive effect, producing a profile that anti-correlates with those of *S. cerevisiae* deletions mutants in the same pathway. Scenarios on which both behaviors occurred could also be imagined. We further expected that the expression of some, but not all, of *C. neoformans* genes would produce profiles that would allow us to develop experimentally testable hypothesis for gene function.

**Figure 2:**
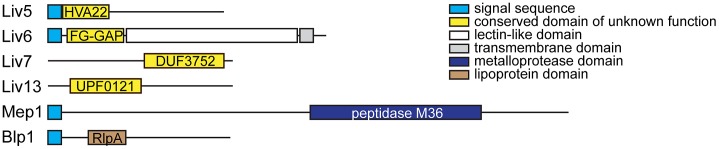
*C. neoformans* bait genes. Schematics of the Liv5, Liv6, Liv7, Liv13, Mep1, and Blp1 proteins. Detectable motifs are shown.

We focused on six *C. neoformans* genes (Figure 2 and Table S1), four of which (*LIV5*, *LIV6*, *LIV7*, and *LIV13*) our previously work identified as necessary for growth in a murine infection model [5]. Two others, *BLP1* and *MEP1*, are targets of Gat201 [22], a master transcriptional regulator of virulence [5], [22]. Blp1 is important for *C. neoformans* to evade phagocytosis by macrophages. Four of these genes (*LIV6*, *LIV7*, *MEP1*, and *BLP1*) lack *S. cerevisiae* orthologs. Several contain conserved domains identified by BLAST [23], but the function of these domains are poorly understood (Figure 2 and Table S1). The application of PHYRE, a threading-based structure prediction algorithm, provided information for only Liv6, which it predicts to be structurally related to a lectin [24].

We generated cross-species genetic interaction profiles using *S. cerevisiae* strains carrying two control constructs and six different bait constructs: *pGPD* (promoter-only control), *pGPD-GFP* (nonspecific protein control), *pGPD-LIV5*, *pGPD-LIV6*, *pGPD-LIV7*, *pGPD-LIV13*, *pGPD-MEP1*, and *pGPD-BLP1*. To ensure reasonable expression levels (see Materials and Methods) used the strong *GPD1* promoter because the *C. neoformans* genome is GC-rich [25] compared to *S. cerevisiae* [26], which is anticipated to inhibit protein translational efficiency due to differences in codon usage and an increased propensity to form inhibitor RNA structures [27]. We calculated Pearson correlations (correlation score) to compare cross-species genetic interaction profiles with the previously described genetic interaction profiles of produced by crosses of 1712×3885 *S. cerevisiae* gene deletions [19]. To avoid potentially spurious correlations, we filtered out correlations with *S. cerevisiae* deletions whose profiles yielded significant correlations with either of the two control baits. Significance testing revealed that correlations with a value of greater than 0.08 are highly significant (P<0.001, two-tailed test, Bonferroni-corrected for multiple hypothesis testing). Quantile-quantile plots of the correlations with *S. cerevisiae* deletions versus standard normal quantiles revealed outliers on one or both tails for all baits (Figure S1). We focused on correlations that departed from the mean by at least three standard deviations (|Z|>3). This conservative strategy yielded from 2–15 hits, depending on the bait (Table 1). The profile of *LIV7* displays the largest number hits, and their identities strongly points to a role in Golgi transport, a prediction whose validation via experiments in *C. neoformans* is described below. The *LIV6* profile correlates positively and negatively with two *S. cerevisiae* genetic profiles, those of deletions in *SYN8* and *ECM21*, respectively. Strikingly, both genes play a role in endosome transport and/or fusion [28], [29], predicting a role for Liv6 in these processes in *C. neoformans*. Support for this prediction via experiments in *C. neoformans* is also described in this paper. Several other profile hits were obtained, but have yet to validated. The Liv5 profile correlates with those of deletions affecting the cell cycle and autophagy [30]–[32] and the Liv13 profile negatively correlates with the genetic profiles of knockouts involved in alleviating protein folding stress [33]–[35]. The profile of the *MEP1* metalloprotease correlates with that of a knockout in a *S. cerevisiae* metalloprotease of a different family, *YBR075W* [23], as well as proteins involved in nucleotide and RNA metabolism. Finally, the genetic interaction profile of the *S. cerevisiae* strain expressing Blp1 positively correlates with that of the deletion of an *S. cerevisiae* gene, *ETR1*, that has a role in fatty acid synthesis. This observation may be related to the Rare Lipoprotein A (RlpA) domain prediction for Blp1 (Table S1).

**Table 1:** *S. cerevisiae* genes whose knockouts significantly correlate with *C. neoformans* bait genes.

*BAIT*	*Yeast HIT*	Z	Correlation	Name	Gene Function (from yeastgenome.org)
*LIV7*	YOR115C	9.2	0.31	*TRS33*	One of 10 subunits of the transport protein particle (TRAPP) complex of the cis-Golgi which mediates vesicle docking and fusion; involved in endoplasmic reticulum (ER) to Golgi membrane traffic
*LIV7*	YOL018C	4.2	0.143	*TLG2*	Syntaxin-like t-SNARE that forms a complex with Tlg1p and Vti1p and mediates fusion of endosome-derived vesicles with the late Golgi; binds Vps45p, which prevents Tlg2p degradation and also facilitates t-SNARE complex formation; homologous to mammalian SNARE protein syntaxin 16 (Sx16)
*LIV7*	YKR020W	3.6	0.123	*VPS51*	Component of the GARP (Golgi-associated retrograde protein) complex, Vps51p-Vps52p-Vps53p-Vps54p, which is required for the recycling of proteins from endosomes to the late Golgi; links the (VFT/GARP) complex to the SNARE Tlg1p
*LIV7*	YLR306W	3.4	0.116	*UBC12*	Enzyme that mediates the conjugation of Rub1p, a ubiquitin-like protein, to other proteins; related to E2 ubiquitin-conjugating enzymes
*LIV7*	YOL052C	3.4	0.11	*SPE2*	S-adenosylmethionine decarboxylase, required for the biosynthesis of spermidine and spermine; cells lacking Spe2p require spermine or spermidine for growth in the presence of oxygen but not when grown anaerobically
*LIV7*	YDR096W	3.1	0.105	*GIS1*	JmjC domain-containing histone demethylase and transcription factor; involved in expression of genes during nutrient limitation
*LIV7*	YMR307W	3	0.102	*GAS1*	Beta-1,3-glucanosyltransferase, required for cell wall assembly and also has a role in transcriptional silencing; localizes to the cell surface via a glycosylphosphatidylinositol (GPI) anchor; also found at the nuclear periphery
*LIV7*	YGR143W	−3.7	−0.121	*SKN7*	Protein involved in sphingolipid biosynthesis; type II membrane protein with similarity to Kre6p
*LIV7*	YKL149C	−3.4	−0.112	*DBR1*	RNA lariat debranching enzyme, involved in intron turnover; required for efficient Ty1 transposition
*LIV7*	YOL061W	−3.4	−0.11	*PRS5*	5-phospho-ribosyl-1(alpha)-pyrophosphate synthetase, synthesizes PRPP, which is required for nucleotide, histidine, and tryptophan biosynthesis; one of five related enzymes, which are active as heteromultimeric complexes
*LIV7*	YNL049C	−3.2	−0.106	*SFB2*	Component of the Sec23p-Sfb2p heterodimer of the COPII vesicle coat, required for cargo selection during vesicle formation in ER to Golgi transport; homologous to Sec24p and Sfb3p
*LIV7*	YOL064C	−3.2	−0.105	*MET22*	Bisphosphate-3′-nucleotidase, involved in salt tolerance and methionine biogenesis; dephosphorylates 3′-phosphoadenosine-5′-phosphate and 3′-phosphoadenosine-5′-phosphosulfate, intermediates of the sulfate assimilation pathway
*LIV7*	YDL213C	−3.2	−0.104	*NOP6*	rRNA-binding protein required for 40S ribosomal subunit biogenesis; contains an RNA recognition motif (RRM); hydrophilin essential to overcome the stress of the desiccation-rehydration process; NOP6 may be a fungal-specific gene as no homologs have been yet identified in higher eukaryotes
*LIV7*	YPR200C	−3.1	−0.1	*ARR2*	Arsenate reductase required for arsenate resistance; converts arsenate to arsenite which can then be exported from cells by Arr3p
*LIV7*	YLR040C	−3	−0.099		Protein of unknown function; localizes to the cell wall; predicted to be a GPI-attached protein
*LIV6*	YAL014C	3.3	0.087	*SYN8*	Endosomal SNARE related to mammalian syntaxin 8
*LIV6*	YBL101C	−3.3	−0.091	*ECM21*	Protein involved in regulating the endocytosis of plasma membrane proteins
*LIV6*	YBR215W	−3.2	−0.087	*HPC2*	Subunit of the HIR complex, a nucleosome assembly complex involved in regulation of histone gene transcription
*LIV5*	YKR019C	3.6	0.097	*IRS4*	EH domain-containing protein involved in regulating phosphatidylinositol 4,5-bisphosphate levels and autophagy; Irs4p and Tax4p bind and activate the PtdIns phosphatase Inp51p; Irs4p and Tax4p are involved in localizing Atg17p to the PAS
*LIV5*	YBR195C	3.4	0.091	*MSI1*	Subunit of chromatin assembly factor I (CAF-1Msi1p localizes to both nucleus and cytoplasm and has an independent role as a negative regulator of the RAS/cAMP pathway via sequestration of Npr1p kinase
*LIV5*	YBR057C	−4.1	−0.11	*MUM2*	Cytoplasmic protein essential for meiotic DNA replication and sporulation; interacts with Orc2p, which is a component of the origin recognition complex
*LIV5*	YKL030W	−3.4	−0.092		dubious; overlaps with MAE1 (Mitochondrial malic enzyme, catalyzes the oxidative decarboxylation of malate to pyruvate, which is a key intermediate in sugar metabolism and a precursor for synthesis of several amino acids)
*LIV13*	YBR169C	−4.1	−0.116	*SSE2*	Member of the heat shock protein 70 (HSP70) family; may be involved in protein folding; localized to the cytoplasm; highly homologous to the heat shock protein Sse1p
*LIV13*	YKL075C	−3.8	−0.108		unknown; proposed to be involved in resistance to streptozotocin and camptothecin
*LIV13*	YBL049W	−3.5	−0.098	*MOH1*	Protein of unknown function, has homology to kinase Snf7p; not required for growth on nonfermentable carbon sources; essential for survival in stationary phase
*LIV13*	YBR181C	−3.1	−0.087	*RPS6B*	Protein component of the small (40S) ribosomal subunit; identical to Rps6Ap and has similarity to rat S6 ribosomal protein
*MEP1*	YAR015W	6.4	0.175	*ADE1*	N-succinyl-5-aminoimidazole-4-carboxamide ribotide (SAICAR) synthetase, required for ‘de novo’ purine nucleotide biosynthesis; red pigment accumulates in mutant cells deprived of adenine
*MEP1*	YKL009W	4.5	0.122	*MRT4*	Protein involved in mRNA turnover and ribosome assembly, localizes to the nucleolus
*MEP1*	YPR114W	3.5	0.094		unknown
*MEP1*	YKR019C	3.4	0.092	*IRS4*	EH domain-containing protein involved in regulating phosphatidylinositol 4,5-bisphosphate levels and autophagy; Irs4p and Tax4p bind and activate the PtdIns phosphatase Inp51p; Irs4p and Tax4p are involved in localizing Atg17p to the PAS
*MEP1*	YBR074W	3.2	0.088		unknown; putative metalloprotease
*MEP1*	YBR119W	−4.2	−0.115	*MUD1*	U1 snRNP A protein, homolog of human U1-A; involved in nuclear mRNA splicing
*MEP1*	YKR055W	−3.5	−0.095	*RHO4*	Non-essential small GTPase of the Rho/Rac subfamily of Ras-like proteins, likely to be involved in the establishment of cell polarity
*BLP1*	YBR026C	3.1	0.085	*ETR1*	2-enoyl thioester reductase, member of the medium chain dehydrogenase/reductase family; localized to in mitochondria, where it has a probable role in fatty acid synthesis
*BLP1*	YKL166C	3	0.083	*TPK3*	cAMP-dependent protein kinase catalytic subunit; promotes vegetative growth in response to nutrients via the Ras-cAMP signaling pathway; partially redundant with Tpk1p and Tpk2p; localizes to P-bodies during stationary phase

*C. neoformans* bait gene (column 1), *S. cerevisiae* ORF that shows significant correlation (column 2), Z-score (column 3), correlation score (column 4), *S. cerevisiae* gene name (column 5), and *S. cerevisiae* gene function (from the *Saccharomyces Genome Database* at yeastgenome.org) [78] (column 6).

### *LIV7* cross-species genetic interaction profile suggests a role in Golgi transport

**Figure 3:**
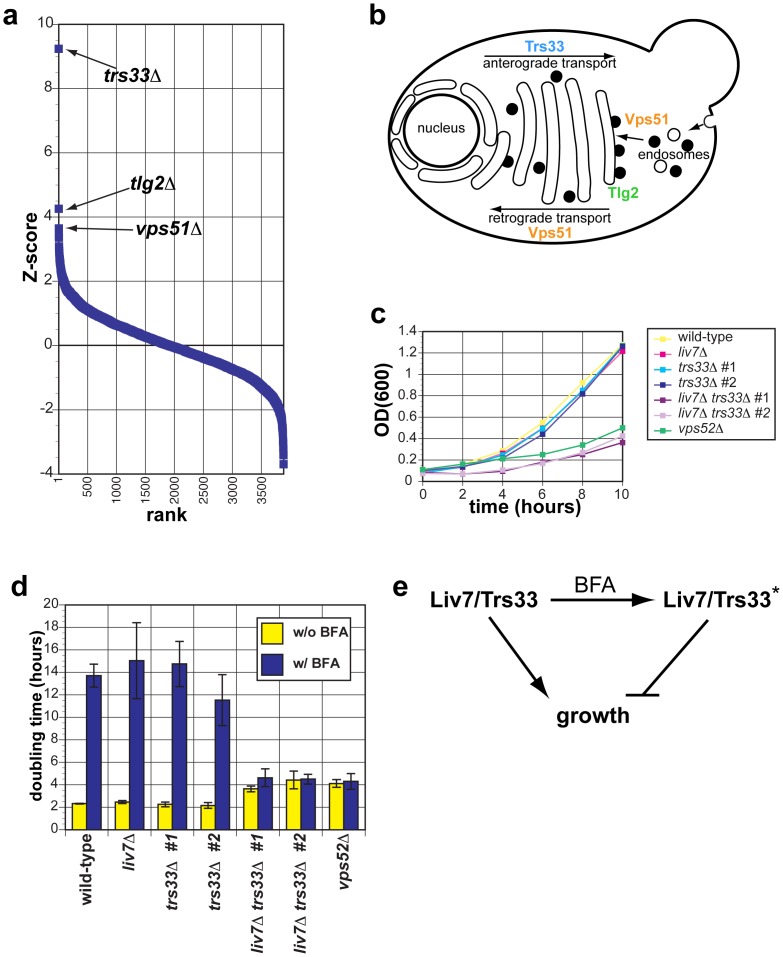
The genetic interaction profile of the *C. neoformans* gene *LIV7* accurately predicts that Liv7 acts within the ER/Golgi in *C. neoformans*. A) Pearson correlations between the genome-wide genetic interaction profiles of *pGPD-LIV7* (blue) with the published genome-wide interaction profiles of *S. cerevisiae* knockout mutants [19]. See Table 1. B) Subcellular roles of *S. cerevisiae* proteins whose deletion profiles correlate with that of *C. neoformans LIV7*. Trs33 is a member of the TRAPP complex and involved in vesicle transport within the Golgi [37]. Tlg2 is a t-SNARE involved in vesicle fusion in the ER/Golgi [39]. Vps51 is one of four members of the GARP complex (Vps51–54) that is involved in endosome-to-Golgi transport and retrograde transport within the Golgi [38]. C) Growth curves of *C. neoformans* mutants in yeast nitrogen base (YNB) at 30°C. OD_600_ was measured every two hours over the course of the experiment. Representative data from three experiments are shown. D) Proliferation analysis. Shown is the doubling time (y-axis) of wild-type, *liv7*Δ, *trs33*Δ, *liv7*Δ *trs33*Δ, and *vps52*Δ strains following treatment with 40 µg/ml Brefeldin A (BFA). Data shown are the average of three experiments and error bars represent the standard deviation and p-values were calculated using Student's t-test. E) Model to explain resistance of *liv7*Δ*trs33*Δ cells to BFA treatment. Without BFA treatment, Liv7 or Trs33 alone promotes growth. With BFA treatment, cells convert to a state in which either Liv7 or Trs33 inhibits growth. This genetic behavior is analogous to that of the *S. cerevisiae* MAP kinase Kss1, which is converted from an inhibitor of filamentous growth to an activator via phosphorylation by the upstream MAP kinase Ste7 [43].

Liv7 (Figure 2) is a 330-residue protein that contains a DUF3752 domain, which is annotated as a conserved domain of unknown function [36]. The profile of the *S. cerevisiae* strain expressing *LIV7* displays the strongest three positive correlations with the published genetic interaction profiles of *S. cerevisiae* gene deletions *trs33*Δ, *tlg2*Δ, and *vps51*Δ (Figure 3A). Strikingly, all three of these genes function in transport events that involve the Golgi apparatus (Figure 3B). Trs33 is one of two nonessential subunits of the TRAPP complex, an essential vesicle tethering complex involved in ER-to-Golgi transport [37]. Vps51 is a member of the GARP complex, another vesicle tethering complex that promotes endosome-to-Golgi transport and retrograde transport within the Golgi [38]. Tlg2 is a t-SNARE that is important vesicle fusion within the Golgi [39]. These data make a strong prediction that the function of the unannotated Liv7 protein is in transport events involving the Golgi apparatus. Below we describe experiments in *C. neoformans* that support this prediction and additional follow-up experiments that led us to find that the Liv7 protein is required for the suppression mannose exposure on the cell surface and the suppression of mannose-dependent phagocytosis by mammalian macrophages.

### *LIV7* and *TRS33* interact genetically in *C. neoformans*

Given that Trs33 is a nonessential subunit of TRAPP, we anticipated that if Liv7 functions to promote TRAPP function in *C. neoformans*, that its gene deletion mutation should display a synthetic lethal or synthetic sick phenotype with a deletion of *TRS33* in *C. neoformans*. We tested this prediction by creating single and double targeted knockouts of *LIV7* and an ortholog of *TRS33* we identified in the *C. neoformans* genome. We found that wild-type, *liv7*Δ, and *trs33*Δ strains all grow at approximately the same rate, with a doubling time of two hours (Figure 3C. In contrast, the *liv7*Δ *trs33*Δ double mutant cells display a severe growth defect, having a doubling time of four hours (Figure 3C). These data demonstrate that *LIV7* and *TRS33* interact genetically in *C. neoformans*, as inferred from analysis of the cross-species genetic interaction profiles described above. We also constructed a deletion in the gene coding for a member of the *C. neoformans* GARP complex, Vps52 (we were unable to delete the *C. neoformans VPS51* gene), and found that it displayed a growth phenotype similar to that of the *liv7*Δ *trs33*Δ double mutant.

### Liv7 and Trs33 act redundantly in ER–Golgi function in *C. neoformans*

We next tested the hypothesis that *LIV7* functions in the ER-Golgi system by using a chemical biology approach that takes advantage of the small molecule Brefeldin A (BFA). BFA is a fungal secondary metabolite that inhibits eukaryotic Sec7-family guanine nucleotide exchange factors that are involved in vesicle transport and themselves localize to the membranes of the ER and Golgi apparatus [40]–[42]. BFA blocks anterograde transport from the ER to the Golgi, fusion of ER and Golgi compartments, and loss of Golgi apparatus itself [40], [41]. We grew strains with and without a growth-inhibitory, sublethal concentration (40 µg/ml) of BFA (Figure 3D). Wild-type, *liv7*Δ, and *trs33*Δ show identical responses to BFA: a sharp increase in doubling time from two hours to over 12 hours (p≤0.01) (Figure 3D). *liv7*Δ *trs33*Δ mutants, which already exhibit slow growth (p≤0.01), do not show any further increase in their four-hour doubling time. The resistance to BFA exhibited by *liv7*Δ *trs33*Δ double mutants demonstrates that either Liv7 or Trs33 function is required for BFA to inhibit cell growth (Figure 3D). These data could be explained if Liv7 and Trs33 have a severe defect in the assembly and/or function of the Golgi apparatus (which we show to be the case below). In this scenario, the growth rate of such cells would thus not be affected by BFA since the have greatly reduced the target organelle most strongly affected by the drug. A more formal statement of such a model would be that in the absence of BFA, Liv7 and Trs33 redundantly promote growth (via a role in Golgi biogenesis), but in the presence of the drug, cells convert to a state in which either Liv7 or Trs33 inhibits growth (Figure 3E). This genetic behavior is analogous to that of the *S. cerevisiae* MAP kinase Kss1, which is converted from an inhibitor of filamentous growth to an activator via phosphorylation by the upstream MAP kinase Ste7 [43]. The *vps52*Δ mutant also displays resistance to BFA (Figure 3D).

**Figure 4:**
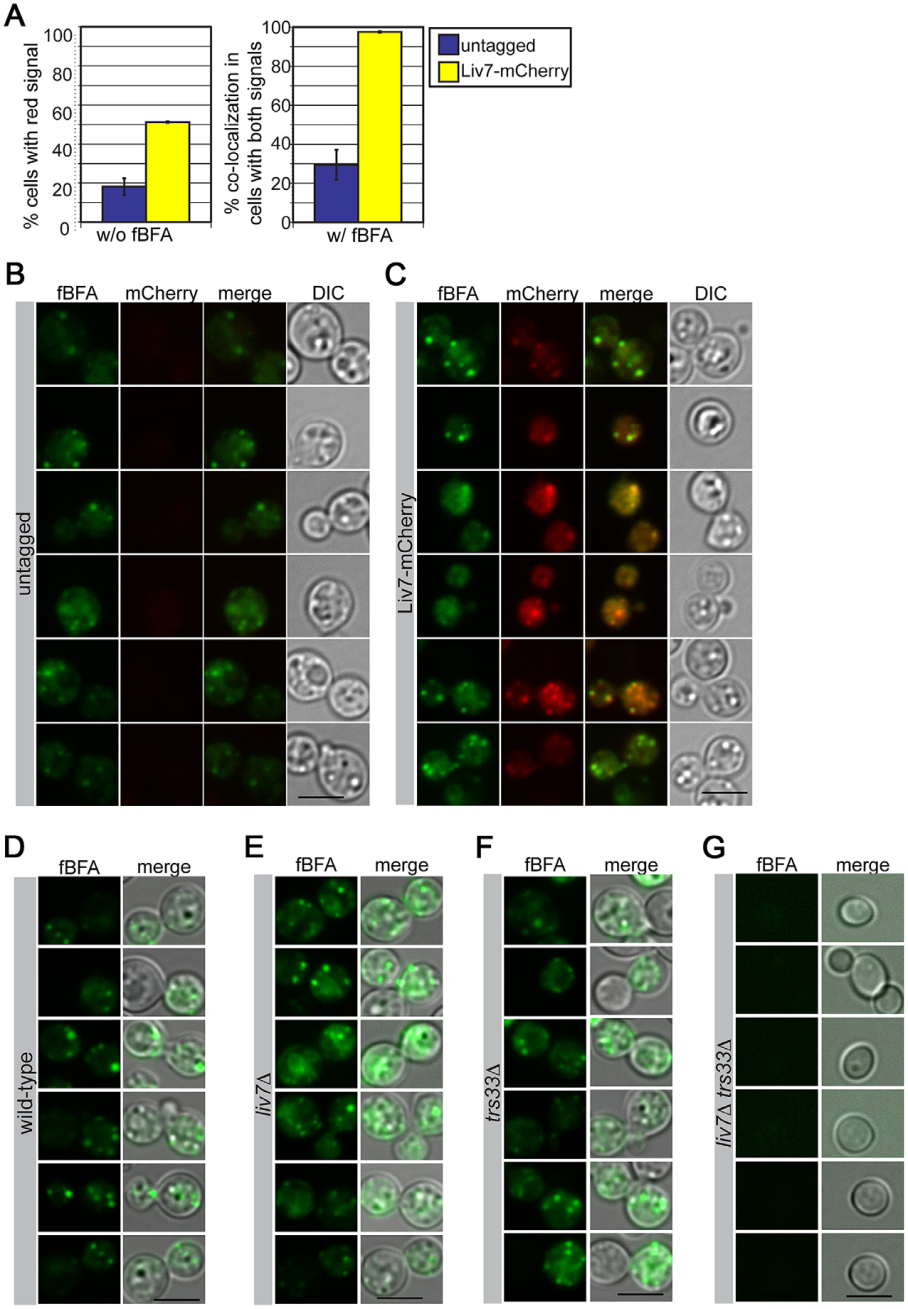
Liv7 localizes to the ER/Golgi in *C. neoformans*. A) Localization analysis. Shown are mCherry signals of cells grown under tissue culture conditions (left) (DMEM, 5% CO_2_, 37°C, without shaking). The untagged control population (blue) shows mCherry signal in less than 20% of cells, whereas mCherry signal is visible in ∼50% of Liv7-mCherry positive cells (yellow). We then stained these same strains with BODIPY-labeled fluorescent BFA (fBFA; green channel; localizes to the ER/Golgi [44]). Experiments were performed three times, 100 cells counted per sample, and data shown are the averages of three experiments. Error bars represent that standard deviation and p-values were calculated using Student's t-test. Scale bars are 5 µm. B) Untagged control cells stained with fBFA. 50 ms exposure. C) Liv7-mCherry cells stained with fBFA. 50 ms exposure. D–G) fBFA-staining of wild-type, *liv7*Δ, *trs33*Δ, and *liv7*Δ *trs33*Δ cells.

To further test the hypothesis that Liv7 functions in the Golgi, we examined the colocalization of an mCherry-tagged version of Liv7 with compartment markers. The levels of Liv7 protein are low and we could not detect it by Western or microscopy under yeast culture conditions (data not shown). However, under the same tissue culture conditions we use to study pathogen phagocytosis (DMEM, 5% CO_2_, without shaking), we observed a punctate Liv7-mCherry signal that was well above background signal observed in an untagged control strain (Figure 4A–4C). To label the ER and Golgi, we briefly incubated cells with a fluorescent derivative of Brefeldin A (fBFA) [44] at sub-inhibitory concentrations (0.5 µg/ml for 40 min, 80-fold less than the minimal inhibitory concentration). To confirm that the compound was labeling the anticipated compartments, we stained a *C. neoformans* strain with fBFA carrying a mCherry-tagged version of the conserved Erd2 protein, which is found in both ER and Golgi compartments [45] and found that the fBFA signal colocalizes with the Erd2 signal (Figure S2). Importantly, the Liv7-mCherry colocalizes with the fBFA signal. The respective puncta co-localize in almost 100% cells that display signals for both fluorophores (Figure 4A–4C). As a control, we stained mitochondria with MitoTracker did not observe co-localization with Liv7-mCherry signal (Figure S3).

To test whether mutations *LIV7* and *TRS33* impact the formation of the ER and Golgi we stained single and double mutants with fBFA. Wild-type, *liv7*Δ, and *trs33*Δ strains showed similar cytoplasmic punctate staining (Figure 4D–4G). However, *liv7*Δ *trs33*Δ mutants did not exhibit detectable fBFA staining (Figure 4G), consistent with a severe defect in organelle formation. These data show that Liv7 is important in promoting organelle formation in cells lacking Trs33. Together with the impact of the mutants on BFA sensitivity and the colocalization of Liv7 with fBFA, these observations provide strong evidence for a role for Liv7 in Golgi function.

### Liv7 and Trs33 suppress PAMP exposure in *C. neoformans*

**Figure 5:**
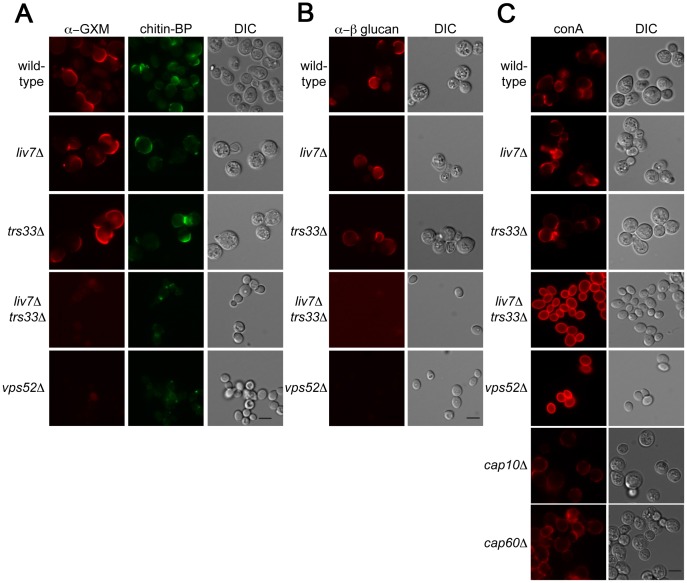
Lectin staining of surface of *liv7*Δ *trs33*Δ cells reveals a role for Liv7/Trs33 in PAMP shielding. A) GXM (left) and chitin (middle) staining of *C. neoformans* strains grown under tissue culture conditions (DMEM, 5% CO_2_, 37°C, without shaking). Scale bars are 5 µm. B) α-β-glucan staining patterns. C) Concanavalin A (conA) staining patterns. Note that *liv7Δtrs33Δ* and *vps52Δ* mutants display a massive increase in conA staining.

Key functions of the Golgi include the sorting and modification of proteins and the biosynthesis of polysaccharides. The cell surface of microbes often contain pathogen-associated molecular patterns (PAMPs), molecular signatures that are recognized by the mammalian immune system [46]. Previous studies of the human fungal pathogen *Candida albicans* has shown that there are mechanisms by which this pathogen masks PAMPs to order to avoid recognition by neutrophils [47]. To test whether *LIV7* or *TRS33* are involved in PAMP exposure, we examined the cell surface exposure of two well-established fungal PAMPs, mannose and β-glucan. These experiments were performed in tissue culture conditions, which modestly induces production of the *C. neoformans* polysaccharide capsule. In addition, we stained cells for the glucuronoxylomannan (GXM) component of the capsule and as well as the cell wall polysaccharide chitin. We used an antibody to detect glucuronoxylomannan (GXM) component of *C. neoformans* polysaccharide capsule (Figure 5A), the lectin CBP to detect chitin (Figure 4A), an antibody to detect β-glucan (Figure 5B), and the lectin concanavalin A (conA) to detect exposure of mannose (Figure 5C). Wild-type, *liv7*Δ, and *trs33*Δ all showed similar PAMP exposure, with modest staining of β-glucan and mannose under tissue culture growth conditions (Figure 5A–5C). We also observed modest staining using reagents that detect chitin and GXM (Figure 5A–5C).

In contrast, we observed strikingly different results in *liv7*Δ *trs33*Δ double mutant cells and in the *vps52*Δ mutant. Most remarkably, we observed a dramatic increase in mannose exposure in these mutants as measured by conA staining (Figure 5C). In contrast, GXM or β-glucan staining is virtually eliminated (Figure 5A, 5B). The chitin signal is reduced in intensity and localizes to a focus at the cell pole. The increase in conA signal cannot be explained by the lack of capsular GXM in the double mutant, as GXM- and capsule-deficient mutant strains *cap10*Δ [48] and *cap60*Δ [48], [49] do not exhibit this phenotype (Figure 5C). These data suggest that *LIV7* and *TRS33* act redundantly in the transport of molecules required to suppress the exposure of mannose on the cell surface and that the integrity of the GARP complex is also required for this process.

### Liv7 prevents mannose-dependent phagocytosis of *C. neoformans* by macrophages

Mannose and mannoproteins (mannan) are highly immunogenic [50], and, consequently, masking their exposure would be expected to be critical for pathogen evasion of the host immune system. It is well-established that *C. neoformans* evades phagocytosis by macrophages (anti-phagocytosis), the first line of host immune defense, and that this attribute is important for mammalian infection [5], [51]. In prior work, we demonstrated that *C. neoformans* evades phagocytosis by at least two pathways, one requiring capsule production and a second that is independent of capsule production and programmed by the transcriptional regulators Gat201 and Gat204 [5], [22]. Strikingly, mutations that abrogate capsule formation and mutations in the capsule-independent pathway do not result in detectable exposure of mannose or β-glucan on the cell surface, suggesting that these pathways do not act by masking these known PAMPs, even though their exposure would be anticipated to activate phagocytic receptors on macrophages.

**Figure 6:**
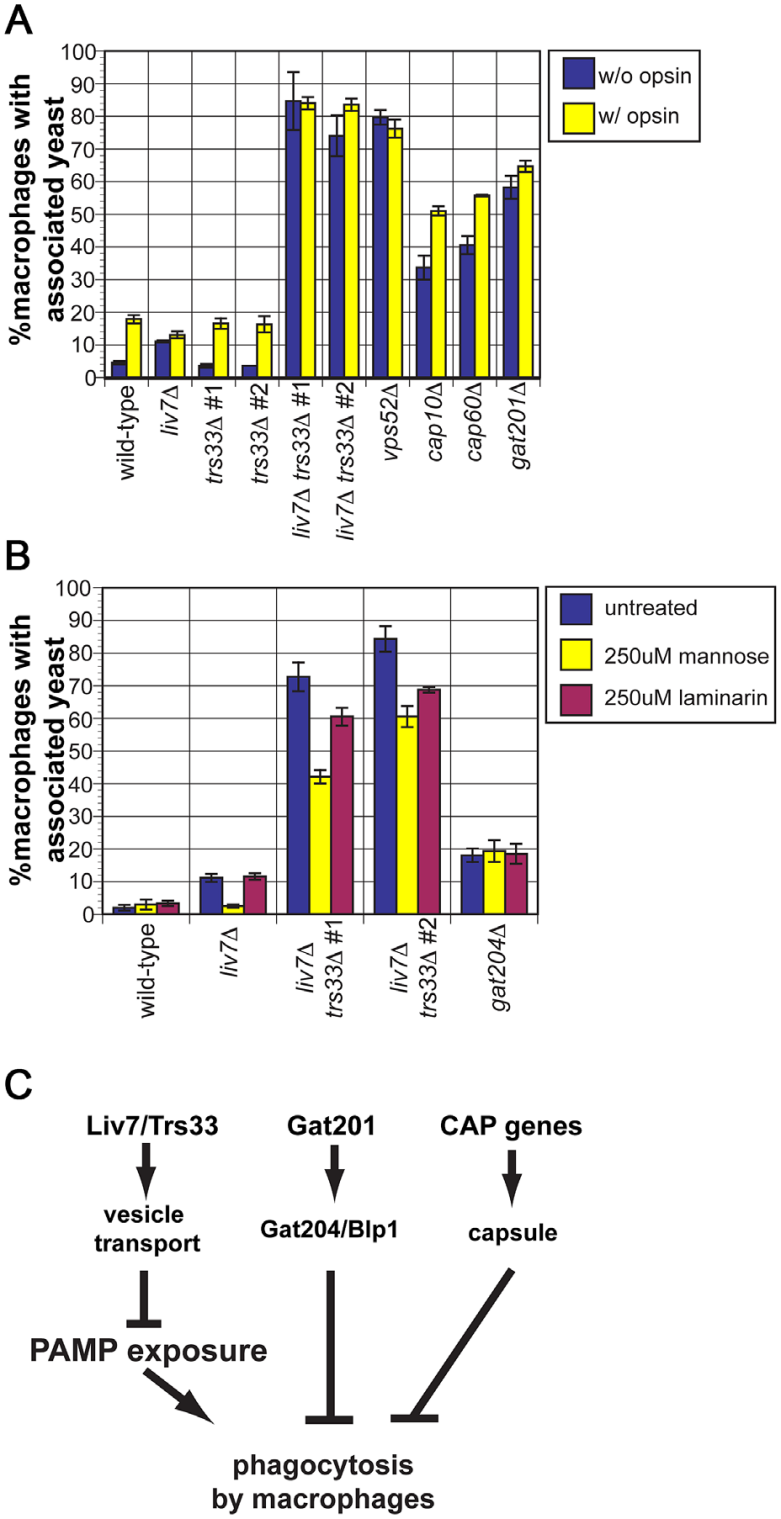
*liv7*Δ but not *trs33*Δ cells are defective in phagocytosis evasion. A) Phagocytosis assays. *C. neoformans* cells were treated either with 1× PBS (unopsonized, blue) or 100% fetal bovine serum (opsonized, yellow) for 30 min, then used to infect RAW264.6 macrophage-like cells at a multiplicity-of-infection of two *C. neoformans* cells to one macrophage. Data shown are the averages of three experiments. Error bars represent that standard deviation and p-values were calculated using Student's t-test. B) Phagocytosis assays. Association of unopsonized *C. neoformans* cells with the addition of control buffer (blue), with 250 µM mannose (yellow), or 250 µM laminarin (purple). Data shown are the averages of three experiments. Error bars represent that standard deviation and p-values were calculated using Student's t-test. C) Model. Together with our previous work, the data described in this paper suggests that there are three parallel mechanisms by which *C. neoformans* evades phagocytosis. First, Liv7 acts in a partially redundant fashion with Trs33 in vesicle transport, a function that prevents exposure of pathogen associated molecular patterns (PAMPs) that are recognized by the immune system and result in phagocytosis of *C. neoformans* by phagocytes. Liv7/Trs33 are not part of the Gat201-Gat204-Blp1 pathway because phagocytosis of *liv7*Δ cells can be competitively inhibited by mannose, whereas phagocytosis of *gat204*Δ cells cannot. The Liv7/Trs33 pathway does not act to suppress phagocytosis via capsule production since capsule-deficient mutants do not display PAMP exposure and are sensitive to opsonization.

Since we observed a dramatic increase in mannose exposure in the *liv7Δ trs33Δ* double mutant, we anticipated that it would display high levels of phagocytosis. To test this, we cultured wild-type, *liv7*Δ, *trs33*Δ, and *liv7*Δ *trs33*Δ *C. neoformans* cells with RAW 264.7 cells, a murine macrophage cell line. To test the potential impact of opsonization, *C. neoformans* strains were treated or not with fetal bovine serum prior to incubation (Figure 6A). Wild-type *C. neoformans* displays a low level of phagocytosis (4% macrophages with associated *C. neoformans* cells) that increased (∼17%) upon opsonization (p≤5×10^−4^). As anticipated from their mannose exposure, *liv7*Δ *trs33*Δ mutants and *vps52*Δ mutants show high levels (∼80%) of phagocytosis regardless of opsonization (p≤2×10^−3^). The lack of anti-phagocytosis activity by *liv7*Δ *trs33*Δ cells and *vps52*Δ cells is not solely due to lack of GXM, as GXM mutants *cap10*Δ and *cap60*Δ show increased association with macrophages (p≤3×10^−3^) but not to the same extent as *liv7*Δ *trs33*Δ cells, and are still sensitive to opsonization (p≤5×10^−3^).

Surprisingly, even though there was no gross increase in mannose exposure in the *liv7*Δ single mutant, it displays a small (11%) but reproducible increase in phagocytosis without opsonization (p≤5×10^−5^) and no further increase with opsonization. In contrast, the *trs33*Δ mutant does not show this phenotype.

The single *liv7*Δ and *trs33*Δ mutants show distinct phagocytosis phenotypes yet the mannose exposure (as determined by conA staining) of both mutants is not distinguishable from wild-type. We hypothesized that *liv7*Δ cells might exhibit an increase in mannose or mannan on their surface not present in *trs33*Δ cells that is too subtle to detect by microscopy-based lectin staining assays. A functional prediction of this hypothesis is that the increase in phagocytosis of the *liv7*Δ mutant should be specifically blocked by an excess of free mannose. We performed phagocytosis assays using unopsonized *C. neoformans* cells and added either soluble mannose (to block recognition of mannose and mannans by macrophage mannose-recognition receptors) or laminarin (a control oligosaccharide that blocks recognition of beta-glucan) [52]. Strikingly mannose, but not laminarin, blocks the increased phagocytosis of *liv7*Δ mutants (p≤10^−3^) (Figure 6B). Mannose addition also partially rescues the anti-phagocytosis defect of *liv7*Δ *trs33*Δ cells (p≤10^−3^). Importantly, this treatment did not impact phagocytic index of *gat204*Δ cells (Figure 6B), a mutant we described previously that produces similar increase in phagocytosis, supporting the view that Liv7 and Gat204 function via distinct mechanisms [22].

### Genetic support for the endosomal role for *LIV6* predicted by its cross-species profile

**Figure 7:**
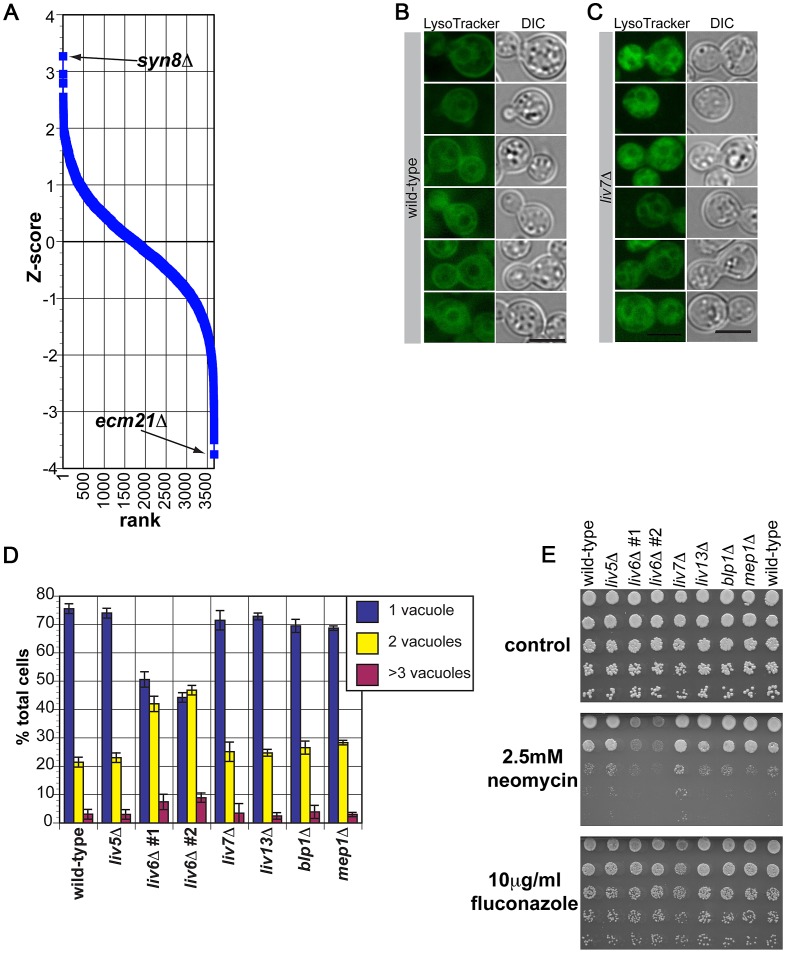
Phenotypes of *C. neoformans liv6*Δ cells are consistent with the endosomal function predicted by cross-species genetic interaction mapping. A) Pearson correlations between the genome-wide genetic interaction profiles of *pGPD-LIV6* (blue) with the published genome-wide interaction profiles of *S. cerevisiae* knockout mutants [19]. See Table 1. B) Wild-type *C. neoformans* cells grown under yeast culture conditions (YNB, 30°C, with shaking) stained with LysoTracker Green. We hypothesize that the dark area surrounded by staining is the vacuole, as it is in *S. cerevisiae* [56]. Fluorescent images were exposed for two seconds and the scale bar represents five microns. C) *liv6*Δ *C. neoformans* cells grown under yeast culture conditions (YNB, 30°C, with shaking) stained with LysoTracker Green. Fluorescent images were exposed for two seconds and the scale bar represents five microns. D) Quantification of the number of “vacuoles” per cell (one, two, or ≥3 putative vacuoles) in LysoTracker-staining *C. neoformans* cells. *liv6*Δ cells but not other mutants show an increase in the number of vacuoles per cell (p≤0.005). Data shown are the averages of three experiments. 200 cells were counted per sample. Error bars represent the standard deviation of three experiments and p-values were calculated using the Student's t-test. E) Growth analysis of wild-type and mutant *C. neoformans* cells on yeast medium (YNB) without drug, with 2.5 mM neomycin, or with 10 µg/ml fluconazole. Cells were spotted at 10^7^ cells/ml in the upper spot and diluted 5-fold in each subsequent spot. Plates were incubated 48 hours at 30°C.

The genetic interaction profile produced by the expression of *LIV6* in *S. cerevisiae* shows positive and negative correlations with the corresponding profiles of the *S. cerevisiae syn8*Δ and *ecm21*Δ deletion mutants, respectively (Table 1 and Figure 7A). These genes act in endosome transport and/or fusion [28], [29], a process that mediates transport from either the plasma membrane or the late Golgi to the vacuole [53]. These correlations predict that Liv6 participates in endosomal functions in *C. neoformans*.

We first tested this prediction by assessing the impact of Liv6 on vacuole number. *S. cerevisiae* genes involved sorting to the vacuole include those that function in endosome biology and often impact vacuole number and morphology [54]–[56]. Vacuoles can be detected by staining with LysoTracker Green (Invitrogen), a dye that is taken up by the cell during endocytosis and fluoresces in acidified compartments, including endosomal vesicles, and typically strains the outer rims of vacuoles. Wild-type *C. neoformans* cells grown in yeast culture conditions and strained with LysoTracker show efficient uptake, many internal vesicles, and rim-stained vacuoles (Figure 7B). This pattern is remarkably similar to those reported for *S. cerevisiae* stained with LysoTracker or FM4-64 [56], [57], an older vital stain used to study protein sorting to the vacuole [57]. Strikingly, *liv6*Δ cells consistently exhibit a greater number of vacuoles than wild-type cells (p<0.005) (Figure 7C, 7D). Notably, the *S. cerevisiae* gene *SYN8*, whose deletion mutant's genetic interaction profile displays a positive correlation with the profile produced by *LIV6* expression (Table 1), has been reported to function with another SNARE to promote normal vacuolar morphology [28]. The increase in vacuolar number seen in *liv6*Δ cells is highly specific, as knockout mutant in any of the bait genes did not exhibit a change in vacuole number (Figure 7D).

We next exploited the aminoglycoside antibiotic neomycin, which interferes with eukaryotic endosomal activity by binding phosphytidylinositol phosphates [58], [59] necessary for endosome function [60]. As a consequence, loss-of-function mutations in *S. cerevisiae* genes involved in endosome function [61], [62] are sensitive to neomycin [61]. Supporting a role for Liv6 in endosome function, we found that *C. neoformans liv6*Δ knockout mutants are sensitive to this drug (Figure 7E). Cells lacking *LIV7* display a subtle reproducible neomycin resistance which could be due to altered cell permeability, a characteristic of neomycin-resistant S. *cerevisiae* strains [63], [64]. Knockout mutants in the other bait genes do not display a change in sensitivity to this compound. *liv6*Δ cells do not exhibit a growth defect on fluconazole, suggesting that their growth defect is specific to neomycin. Together, the changes in vacuole number and sensitivity to neomycin in produced by the *liv6*Δ mutation support the prediction from cross-species genetic profiles of a role for Liv6 in the endovacuolar system of *C. neoformans*.

## Discussion

Genetic approaches to understanding mechanisms of virulence in human fungal pathogens can efficiently identify genes necessary for pathogens to cause disease. However, a key roadblock to progress is the lack of tools that can help define the function of a gene product when its predicted sequence offers few clues to its biochemical function, a common occurrence. We described here a case study of a cross-species genetic interaction profiling approach to develop testable hypotheses for the function of fungal virulence factors of unknown function. Notably, this proof-of-principle study shows that the approach can provide information on fungal pathogenicity factors that lack *S. cerevisiae* orthologs. Although many studies have used *S. cerevisiae* to investigate the function of foreign genes [9]–[11], [13], [14], the cross-species genetic interaction profile used here represents an application of quantitative genetic profiling of foreign proteins in *S. cerevisiae* coupled with comparison to recently described genetic map of *S. cerevisiae* [19] to the problem of annotation of fungal virulence factors. Because *S. cerevisiae* is a fungus, we anticipate that this approach may be particularly useful for fungal genes but that the method may also find utility in the study of bacterial and viral proteins that impact conserved intracellular processes in eukaryotic host cells.

Our approach involves expression in *S. cerevisiae* of cDNAs encoding *Cryptococcus neoformans* virulence factors identified in systematic genetic screens; the generation of genetic profiles by assessing the effect of *C. neoformans* gene expression in the context of each nonessential *S. cerevisiae* deletion mutants; and, correlation analysis with the existing database of genetic interactions to develop testable functional hypotheses. As mentioned above, one mechanism whereby expression of a *C. neoformans* gene could produce impact *S. cerevisiae* would be “dominant-negative” effect thereby inhibiting the activity of an *S. cerevisiae* pathway. Our results with *LIV7* in both *S. cerevisiae* and *C. neoformans* are consistent with this scenario. The expression of *LIV7* in *S. cerevisiae* produces a profile that correlates with that of the *S. cerevisiae trs33*Δ deletion mutant, but in *C. neoformans*, the *liv7*Δ mutation produces a synthetic phenotype with the *trs33*Δ mutation. Alternatively, expression of a *C. neoformans* gene product could act in a “dominant-active” fashion to increase the activity of a pathway which might result in a negative correlation with the profile of a gene deletion in the corresponding pathway. With Liv6, we observed both positive and negative correlations that led us to test a role in endosome function. Although we have focused on the extensive deletion mutant genetic interaction dataset [19], comparisons of the cross-species profiles generated here with genetic interaction profiles produced using chemicals [65], [66] and/or overexpressed genes [67], [68] will likely be equally useful as these approaches are applied on a larger scale. Thus, the analysis of correlations between cross-species genetic interaction profiles and existing “within-species” genetic interaction profiles offers a tool for generating testable predictions for pathways in which foreign genes operate.

The genetic profiling studies and validation experiments described in this paper provide new information on two *C. neoformans* pathogenicity factors identified previously, Liv7 and Liv6. These proteins lack orthologs in *S. cerevisiae* and lack orthologs of known function in other species. Our studies of Liv7 suggest it functions in Golgi transport in a process that suppresses the exposure of the PAMP mannose on the cell surface (Figure 6C). The increased phagocytosis phenotype of the *liv7*Δ single mutant and its specific suppression by soluble mannose appears specific to *liv7*Δ cells and is specific to mannose versus other carbohydrates (Figure 6B). The anti-phagocytic properties of *C. neoformans* are critical for mammalian infection [5], [22], [69] and the capsule is important for the anti-phagocytosis activity of opsonized *C. neoformans* cells [51]. Our previous work identified a capsule-independent pathway necessary for anti-phagocytosis under unopsonized conditions [22]. The suppression cell surface exposure of PAMP mannose appears to represent a third anti-phagocytosis pathway (Figure 6C) since mannose does not rescue the anti-phagocytic defect of *gat204*Δ cells (Figure 6B), which are defective in the capsule-independent anti-phagocytosis pathway [22]. This argument is supported by the observation that *cap10*Δ and *cap60*Δ cells, which lack GXM [48], [49], do not exhibit increased conA staining (Figure 5C). We suggest that Liv7 is important for mammalian infection [5] because it inhibits macrophage recognition of mannose-containing patterns on the *C. neoformans* cell surface (Figure 6C). Although our studies of Liv6 point to a role in endosome biology that impacts neomycin resistance and vacuole number, understanding how this function relates to its role in pathogen fitness in the host will require further investigation. One possibility is that Liv6 is involved in the endocytic uptake of limiting factors required for proliferation from the host milieu.

One anticipates that functional annotation of fungal virulence factors identified genetically will continue to be a major challenge for the future. The approach described here represents one generic tool that could be applied to this problem on a larger scale. We expect that a substantial number of virulence genes of unknown function in fungal pathogens will impinge on conserved cellular processes and that their genetic profiling in *S. cerevisiae* could therefore yield testable functional predictions in a significant number of cases. The cross-species interaction profiling could also be useful for studying genes from highly virulent pathogens that are difficult to work with due to the requirement for extensive containment.

## Methods

### Generation of *S. cerevisiae* strains

We inserted the *GPD1* promoter region, our *C. neoformans* cDNA of interest, and a NAT resistance marker into the multicloning site of pRS316. For recombination into *S. cerevisiae*, we cut with a restriction enzyme that cleaved within the *URA3* locus, the transformed the linearized vector into *S. cerevisiae* using standard lithium acetate-based transformation techniques. We verified expression of *C. neoformans* genes by extracting total RNA from log-phase *S. cerevisiae* cultures grown at 30°C in YNB, selecting for mRNA, and making cDNA as previously described [70]. Expression of *C. neoformans* genes was verified by qPCR performed as previously described [70].

### Calculating *C. neoformans* gene expression level in *S. cerevisiae* strains

We expressed each *C. neoformans* gene under the *GPD1* promoter and we measured RNA by qRT-PCR (Figure S4). We then measured the levels of *BUD1* mRNA in the same RNA preparation. *BUD1* is a small GTPase expressed at low levels [71]–[73] along with its two co-regulators *BUD2* and *BUD5* [74], [75]. We used published data on the molecules of *BUD1* RNA per cell averaged with co-regulators *BUD2* and *BUD5* [71]–[73] to estimate the number of RNA molecules per cell for *C. neoformans* genes from the ratios in Figure S4, then calculated its rank position compared to other *S. cerevisiae* genes. *BLP1* and *LIV6* were in the lowest 10% of genes with detectable RNA (∼5090 of ∼6580 genes had detectable RNA [71]–[73]). *LIV5* and *LIV7* were in the 10–20^th^ percentile, as were the *BUD* genes. *MEP1* was the best expressed of the *C. neoformans* genes, (∼35^th^ percentile). *LIV13* was expressed based on the increase in *LIV13* primer products with and without RT (data not shown) but not compared to *BUD1*.

### Synthetic Genetic Analysis (SGA) screens

We performed SGA screens as described in Tong et al [16], [17] using a RoToR colony pinning robot (Singer Instruments). All screen plates were scanned on a flatbed scanner with autofocus. We extracted colony size data using the publicly available ScreenMill software [76]. We then adjusted the raw colony size data to control for plate position, edge effects, and slow growth of knockout mutants using the S-score method developed by Collins et al [20]. The final S-scores, one for each double mutant strain, indicate the strength of the genetic interaction (absolute value) and whether the interaction is synthetically sick (negative numbers) or buffered (positive numbers) [20]. We then adjusted S-scores so that they were on a scale between −1.0 and 1.0 and calculated the Pearson correlation between S-scores and ε-scores from Constanzo et al [19]. We calculated p-values of the Pearson correlations by calculating the Z-score of the Pearson correlation for each interaction, then using the Z-score to determine the p-value of each interaction.

### *C. neoformans* genetic manipulations and growth conditions

*C. neoformans* was routinely grown in yeast culture conditions in either YPAD (1% yeast extract, 2% peptone, 2% glucose, 0.015% L-tryptophan, 0.004% adenine) or yeast nitrogen base (YNB) (Difco). Strain construction and genetic manipulation was previously described [5]. Whenever more than one knockout mutant for a single gene is shown, mutants were made by independent transformations. Growth curves (Figure 3A) were performed in YNB at 30°C by taking measurements of OD_600_ every two hours for 10 hrs. The growth curve was repeated three times and representative data are shown. When *C. neoformans* cells were grown in tissue culture conditions, they were first grown overnight to saturation in YNB, then washed once in 1× PBS and resuspended at a density of 1 OD_600_/ml (∼1.7×10^7^ cells/ml) in DMEM, then incubated for the specified amount of time in 5% CO_2_ at 37°C.

### Treatment with Brefeldin A (BFA)

Samples were grown in overnight in YNB at 30°C, then subcultured to OD_600_ = 0.2. BFA or DMSO (-BFA control) was added to each culture and the OD_600_ taken every hour for 10 hr. Doubling time was calculated over the interval from 4–8 hr. The treatment curve was repeated three times and the data shown are averages of the three experiments.

### Imaging of Liv7-mCherry and staining with fluorescent Brefeldin A (fBFA)

Samples were grown overnight in YNB at 30°C, then washed 3× in 1× PBS and resuspended at 1 OD/ml in DMEM, then incubated 16 hr under tissue culture conditions (5% CO_2_, 37°C). Samples were then either imaged (unstained samples) or fBFA (Life Technologies) was added to the medium to a final concentration of 0.5 µg/ml. fBFA samples were incubated 40 min, washed 1× in PBS, then imaged immediately.

### LysoTracker staining

*C. neoformans* cells were grown overnight under yeast culture conditions (yeast nitrogen base (YNB), 30°C with rotation), then subcultured to OD_600_ of ∼0.2 and grown to midlog phase. LysoTracker Green was added to a final concentration of 500 nM and incubated for five minutes with shaking at 30°C. Cells were then harvested and immediately imaged.

### MitoTracker staining

Strains were grown under tissue culture conditions for 12 hr. MitoTracker Green (Invitrogen) was added to a final concentration of 1 µM (from 1 mM stock in DMSO), incubated 30 min at 37°C, then imaged.

### Lectin and antibody staining of cell surface residues

Samples were grown overnight in YNB at 30°C, then washed three times in 1× PBS and resuspended at 1 OD/ml in DMEM, then incubated 16 hr under tissue culture conditions (5% CO_2_, 37°C). Samples were then fixed for 15 min in 4% paraformaldehyde, washed three times in 1× PBS, and then used for staining. To stain with concanavalin A (conA) staining for mannose residues, cells were incubated 5 min in 50 µg/ml Alexa Fluor 594 (Invitrogen), washed once in 1× PBS, then imaged. Samples for staining for chitin and GXM were incubated with αGXM antibody mAb 339 (1∶1000) as previously described [5] and fluorescein-conjugated chitin binding domain (New England Biolabs) (1∶500) for 4 hr, then washed twice in 1× PBS and incubated with TRITC-conjugated donkey anti-mouse secondary antibody (Jackson ImmunoResearch) and fluorescein-conjugated chitin binding domain (1∶500) for 1 hr. Samples were then washed once and imaged using an Axiovert 200 M (Zeiss) microscope running Axiovision software. β-glucan staining was performed using the same procedure as GXM staining but with anti-β-glucan antibody (1∶1000) (Biosupplies Australia).

### Phagocytosis assays

Phagocytosis assays were performed as previously described [5], [22]. RAW264.7 macrophages (2×10^4^ cells/well) were seeded into 96-well tissue-culture treated plates in DMEM medium and allowed to adhere overnight. *C. neoformans* cells grown in YPAD medium were washed three times with PBS, then resuspended to a density of 10^7^ cells/ml in PBS. 200 µl fresh DMEM was added to RAW264.7 cells. 5 µl *C. neoformans* culture (5×10^4^ cells) were then added to each well for a multiplicity of infection of two yeast to one macrophage. Following 24 hr co-incubation, the macrophages were washed three times with PBS to remove unphagocytosed yeast and then fixed with 1% formaldehyde/PBS prior to visualization on an inverted light microscope. Percentage of yeast cell-associated macrophages was determined by counting the number of macrophages with yeast internalized or associated with their cell surface, divided by the number of macrophages counted. At least 200 macrophages were assayed per well, and each strain was tested in triplicate. If performing phagocytosis experiments under opsonizing conditions, *C. neoformans* cells were grown overnight in YNB, washed three times in 1× PBS, resuspended to a density of 10^7^ cells/ml in either fetal bovine serum (opsonized samples; FBS) or 1× PBS (unopsonized samples), incubated for 30 min at 30°C on a shaking platform, washed once in 1× PBS, then resuspended at 10^7^ cells/ml in 1× PBS and used to infect macrophages as above.

## Supporting Information

Figure S1 Q–Q plots of *C. neoformans* bait genes. Plots of the quantiles of the indicated filtered genetic interaction profiles plotted against those of a normal distribution. Observations that lie on the diagonal are indicative of normally-distributed data, whereas tails indicate a deviation from the expectation.
(TIF)

Figure S2 fBFA co-localizes with Erd2-mCherry, confirming that fBFA stains the ER and Golgi compartments. A) Six representative cells expressing Erd2-mCherry and stained with fBFA. Growth and staining procedures were performed as in Figure 3C–3E and experiments were carried out simultaneously. Erd2-mCherry and fBFA are co-localized. Scale bars represent five microns. B) mCherry signal from cells grown under tissue culture conditions (left) (DMEM, 5% CO_2_, 37°C). The untagged control population (blue) shows mCherry signal in less than 20% of cells, whereas mCherry signal is visible in ∼50% of Liv7-mCherry expressing cells (yellow) or ∼65% of Erd2-mCherry expressing cells (purple), demonstrating that both Liv7-mCherry and Erd2-mCherry are visible above background levels. When we stained these strains with fBFA, Erd2-mCherry cells with both mCherry and fBFA signal showed co-localization ∼96% of the time, compared to ∼30% of the time for the untagged control. The co-localization of fBFA and Erd2-mCherry, an ER/Golgi marker [45], demonstrate that fBFA localizes to the ER/Golgi as expected. These data are the complete dataset from Figure 3C. Experiments were performed three times, 100 cells counted per sample, and data shown are the averages of three experiments. Error bars represent that standard deviation.
(TIF)

Figure S3 Mitochondria do not co-localize with Liv7-mCherry or Erd2-mCherry. Example cells of Erd2-mCherry (A) and Liv7-mCherry (B) cells stained with MitoTracker Green. As both mitochondrial and Golgi proteins can appear punctate [79], this serves as a negative control to exclude mitochondrial localization for Liv7.
(TIF)

Figure S4 RT–qPCR of *C. neoformans* bait genes expressed in *S. cerevisiae. C. neoformans* genes RNA levels measured by RT-qPCR and compared to *S. cerevisiae* gene *BUD1*, a GTPase involved.
(TIF)

Table S1 *C. neoformans* bait genes. *C. neoformans* bait genes (column 1); their systematic names (column 2); their *S. cerevisiae* ortholog, if any [23] (column 3); the function of the orthologous *S. cerevisiae* gene (column 4); conserved motifs or domains detected by BLAST [23] (column 5); the function of conserved domains or motifs (column 6); and any functional prediction made by the threading program PHYRE [24] (column 7).
(PDF)
