# Supplementary figures and images for: Approaching the Functional Annotation of Fungal Virulence Factors Using Cross-Species Genetic Interaction Profiling
Source: PLoS Genet. 2012 Dec 27;8(12):e1003168. doi: 10.1371/journal.pgen.1003168 (PMC3531484; doi:10.1371/journal.pgen.1003168)

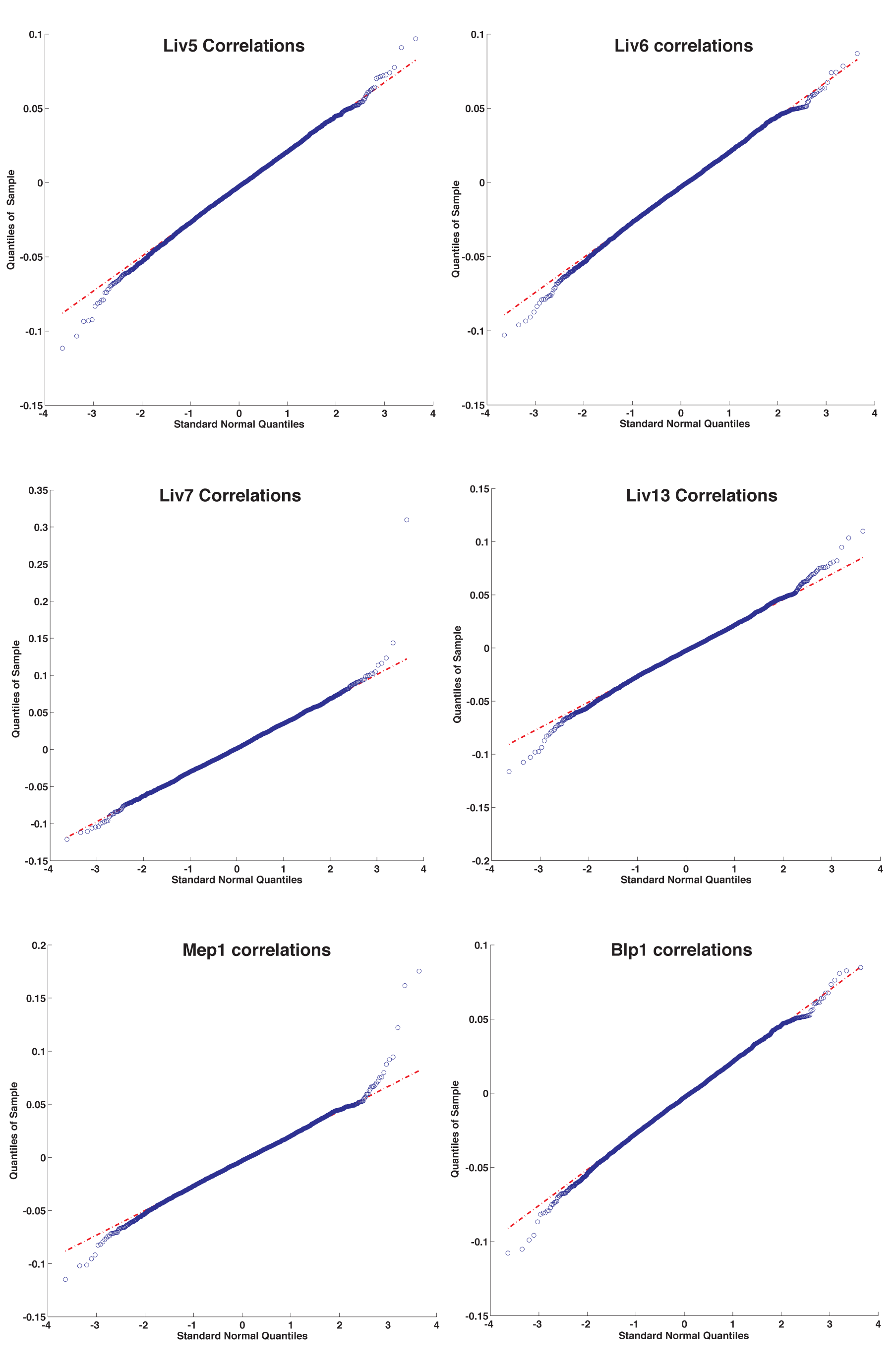

Supplement: Figure S1 — Q–Q plots of C. neoformans bait genes. Plots of the quantiles of the indicated filtered genetic interaction profiles plotted against those of a normal distribution. Observations that lie on the diagonal are indicative of normally-distributed data, whereas tails indicate a deviation from the expectation. (TIF) [file pgen.1003168.s001.tif]

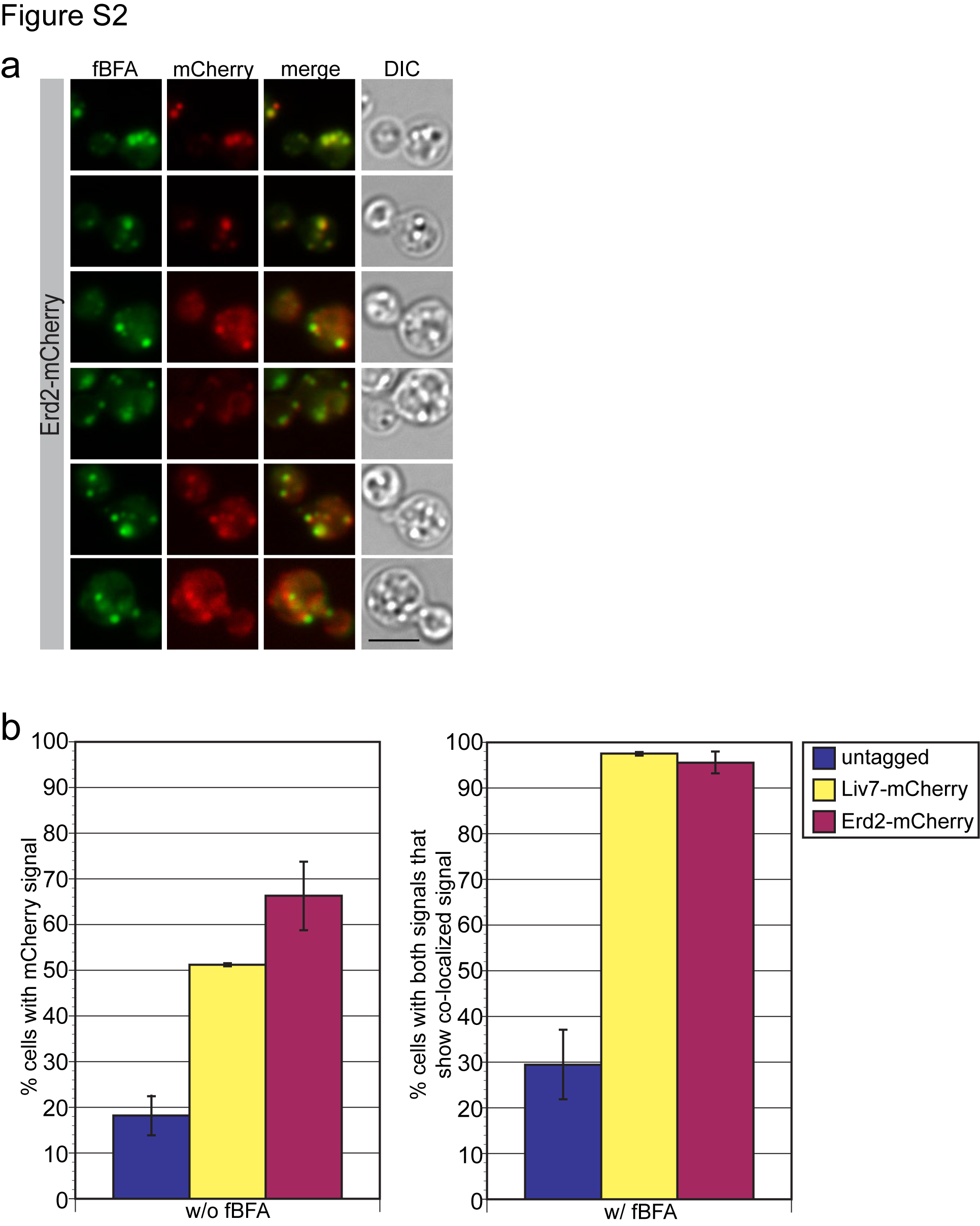

Supplement: Figure S2 — fBFA co-localizes with Erd2-mCherry, confirming that fBFA stains the ER and Golgi compartments. A) Six representative cells expressing Erd2-mCherry and stained with fBFA. Growth and staining procedures were performed as in Figure 3C–3E and experiments were carried out simultaneously. Erd2-mCherry and fBFA are co-localized. Scale bars represent five microns. B) mCherry signal from cells grown under tissue culture conditions (left) (DMEM, 5% CO2, 37°C). The untagged control population (blue) shows mCherry signal in less than 20% of cells, whereas mCherry signal is visible in ∼50% of Liv7-mCherry expressing cells (yellow) or ∼65% of Erd2-mCherry expressing cells (purple), demonstrating that both Liv7-mCherry and Erd2-mCherry are visible above background levels. When we stained these strains with fBFA, Erd2-mCherry cells with both mCherry and fBFA signal showed co-localization ∼96% of the time, compared to ∼30% of the time for the untagged control. The co-localization of fBFA and Erd2-mCherry, an ER/Golgi marker [45], demonstrate that fBFA localizes to the ER/Golgi as expected. These data are the complete dataset from Figure 3C. Experiments were performed three times, 100 cells counted per sample, and data shown are the averages of three experiments. Error bars represent that standard deviation. (TIF) [file pgen.1003168.s002.tif]

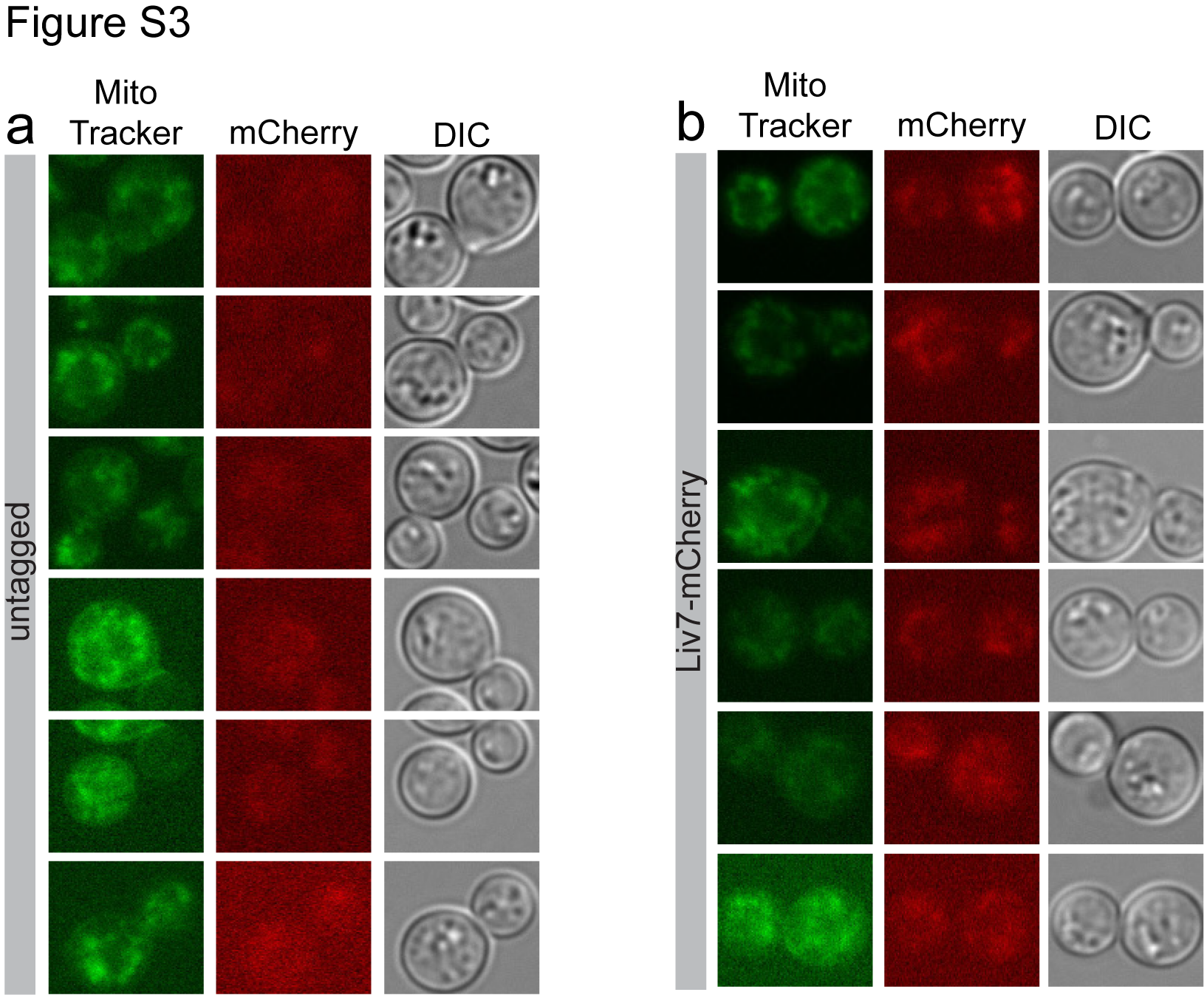

Supplement: Figure S3 — Mitochondria do not co-localize with Liv7-mCherry or Erd2-mCherry. Example cells of Erd2-mCherry (A) and Liv7-mCherry (B) cells stained with MitoTracker Green. As both mitochondrial and Golgi proteins can appear punctate [79], this serves as a negative control to exclude mitochondrial localization for Liv7. (TIF) [file pgen.1003168.s003.tif]

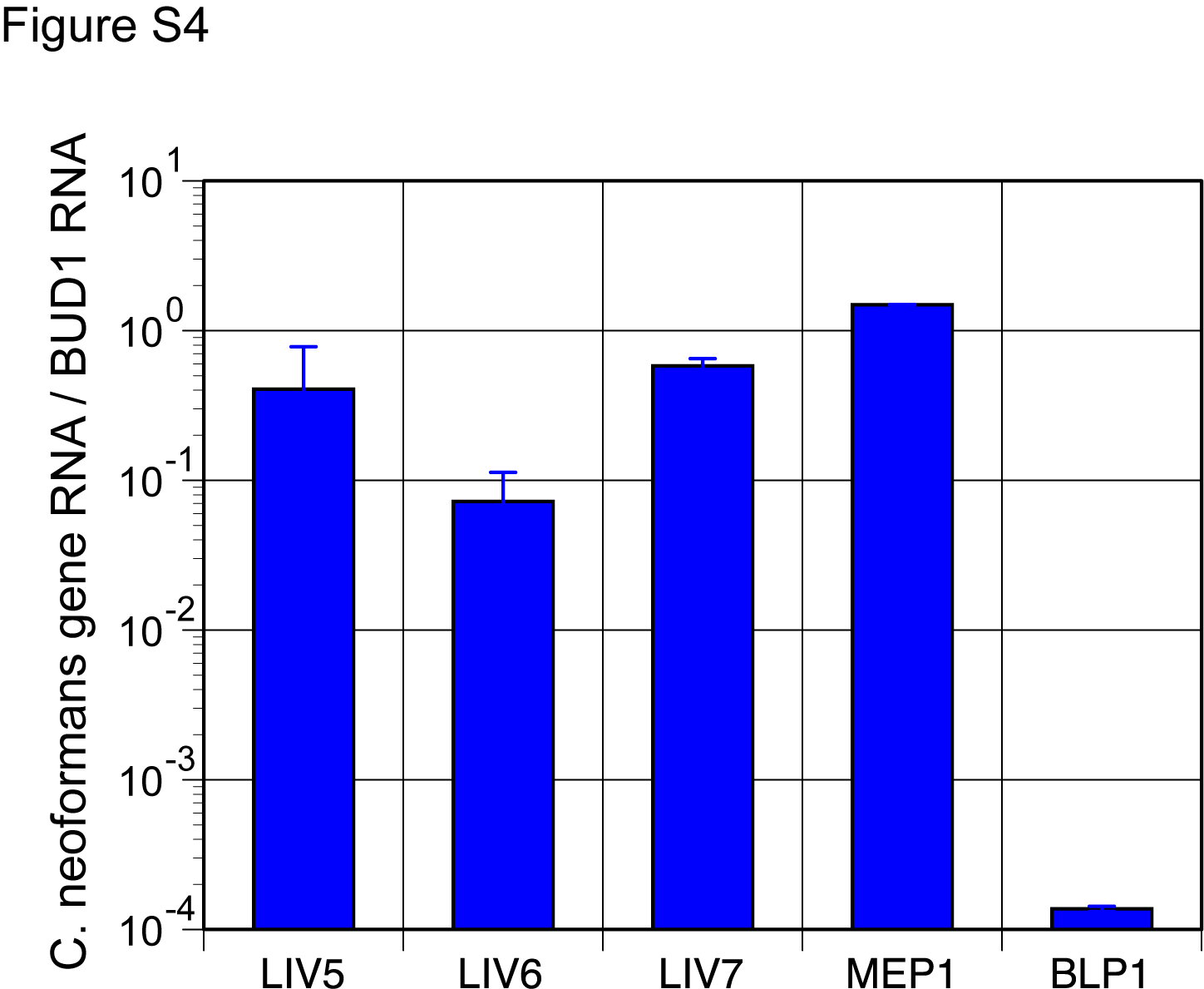

Supplement: Figure S4 — RT–qPCR of C. neoformans bait genes expressed in S. cerevisiae. C. neoformans genes RNA levels measured by RT-qPCR and compared to S. cerevisiae gene BUD1, a GTPase involved. (TIF) [file pgen.1003168.s004.tif]
