# Supplementary material for: Approaching the Functional Annotation of Fungal Virulence Factors Using Cross-Species Genetic Interaction Profiling
Source: PLoS Genet. 2012 Dec 27;8(12):e1003168. doi: 10.1371/journal.pgen.1003168 (PMC3531484; doi:10.1371/journal.pgen.1003168)
Supplement: Table S1 — C. neoformans bait genes. C. neoformans bait genes (column 1); their systematic names (column 2); their S. cerevisiae ortholog, if any [23] (column 3); the function of the orthologous S. cerevisiae gene (column 4); conserved motifs or domains detected by BLAST [23] (column 5); the function of conserved domains or motifs (column 6); and any functional prediction made by the threading program PHYRE [24] (column 7). (PDF) [file pgen.1003168.s005.pdf]

Table S1						
**Gene Name**	**Systematic Name**	***S. cerevisiae* ortholog (E score)**	***S. cerevisiae* gene function**	**Conserved motifs/domains**	**Domain Function**	**PHYRE**
LIV5	CNAG_02409	*YOP1* (0.00021)	ER tubulation	TB2/DP1/HVA22	unknown	none
LIV6	CNAG_07315	none	none	FG-GAP/VCBS	unknown; found in pathogens and integrins	lectin-like
LIV7	CNAG_06464	none	none	DUF3752	unknown	none
LIV13	CNAG_02753	*POM33* (8.3x10^-12)	nuclear pore	UPF0121	unknown; found in ER proteins	transmembrane domains
MEP1	CNAG_04735	none	none	peptidase M36	metalloprotease	none
BLP1	CNAG_06346	none	none	RlpA	lipid binding	none
